# Global Rescue of Defects in HIV-1 Envelope Glycoprotein Incorporation: Implications for Matrix Structure

**DOI:** 10.1371/journal.ppat.1003739

**Published:** 2013-11-14

**Authors:** Philip R. Tedbury, Sherimay D. Ablan, Eric O. Freed

**Affiliations:** Virus-Cell Interaction Section, HIV Drug Resistance Program, Center for Cancer Research, National Cancer Institute, Frederick, Maryland, United States of America; University of Massachusetts Medical School, United States of America

## Abstract

The matrix (MA) domain of HIV-1 Gag plays key roles in membrane targeting of Gag, and envelope (Env) glycoprotein incorporation into virions. Although a trimeric MA structure has been available since 1996, evidence for functional MA trimers has been elusive. The mechanism of HIV-1 Env recruitment into virions likewise remains unclear. Here, we identify a point mutation in MA that rescues the Env incorporation defects imposed by an extensive panel of MA and Env mutations. Mapping the mutations onto the putative MA trimer reveals that the incorporation-defective mutations cluster at the tips of the trimer, around the perimeter of a putative gap in the MA lattice into which the cytoplasmic tail of gp41 could insert. By contrast, the rescue mutation is located at the trimer interface, suggesting that it may confer rescue of Env incorporation via modification of MA trimer interactions, a hypothesis consistent with additional mutational analysis. These data strongly support the existence of MA trimers in the immature Gag lattice and demonstrate that rescue of Env incorporation defects is mediated by modified interactions at the MA trimer interface. The data support the hypothesis that mutations in MA that block Env incorporation do so by imposing a steric clash with the gp41 cytoplasmic tail, rather than by disrupting a specific MA-gp41 interaction. The importance of the trimer interface in rescuing Env incorporation suggests that the trimeric arrangement of MA may be a critical factor in permitting incorporation of Env into the Gag lattice.

## Author Summary

One of the enduring problems in HIV-1 research is the mechanism of incorporation of the viral envelope (Env) glycoprotein into viral particles. Several models have been proposed ranging from an entirely passive process to a requirement for binding of Env by the matrix (MA) domain of the Gag precursor polyprotein. It is clear that specific regions within MA and Env play important roles, as mutations in these domains can prevent Env incorporation. We have identified a point mutation in MA that rescues a broad range of Env-incorporation defective mutations, located both in MA and in Env. Our investigations into the mechanism of rescue have revealed the importance of interactions between MA monomers at a trimeric interface. Our results are consistent with previously published crystallographic models and now provide functional support for the existence of MA trimers in the immature Gag lattice. Furthermore, as the modification of trimer interactions plays a role in the rescue of Env incorporation, we propose that MA trimerization and the organization of the MA lattice may be critical factors in Env incorporation.

## Introduction

Human immunodeficiency virus type 1 (HIV-1), like all replication-competent orthoretroviruses, encodes three main polyproteins – Gag, Pol and Env – which contain determinants necessary for particle assembly, enzymatic functions, and virus entry, respectively. HIV-1 assembly occurs in a series of steps, driven by the Gag precursor protein Pr55^Gag^ (for review, see [1]). HIV-1 Gag is comprised of four domains – matrix (MA), capsid (CA), nucleocapsid (NC) and p6 – and two spacer peptides located between CA and NC, and NC and p6. Pr55^Gag^ is able to form virus-like particles (VLPs) when expressed in cells in the absence of any other viral protein. The MA domain at the N-terminus of Pr55^Gag^ directs cytoplasmic Gag to bind raft-like domains of the plasma membrane (PM) via specific recognition of phosphatidylinositol-4,5-bisphosphate [PI(4,5)P_2_] [2] (for review, [3]). MA binding to PI(4,5)P_2_, as well as Gag oligomerization, triggers a myristyl switch, exposing the myristic acid moiety covalently linked to the amino-terminus of MA [4], [5]. The exposed myristic acid then inserts into the phospholipid bilayer, anchoring Gag to the PM.

In addition to its PM-targeting function, MA is required for the incorporation of the viral Env glycoprotein complex into virions (reviewed in [6], [7]). Env is translated as a polyprotein precursor, gp160, at the endoplasmic reticulum before it traffics to the PM through the Golgi apparatus, where it is cleaved into the mature surface glycoprotein gp120 and transmembrane glycoprotein gp41. The mature Env glycoproteins remain associated as heterotrimers. Gp120 is located entirely on the exterior of the virion and mediates binding to the receptor (CD4) and co-receptors (CXCR4 or CCR5), while gp41 anchors the Env complex in the lipid bilayer and mediates fusion between the viral and target cell membranes. HIV-1 gp41, like the transmembrane glycoproteins of many lentiviruses, possesses a very long cytoplasmic tail (CT). The long gp41 CT, which contains a variety of trafficking motifs (for review see [8]), is required for the incorporation of Env into virus particles during assembly in physiologically relevant cell types such as CD4^+^ T cells and monocyte-derived macrophages (MDMs), although it is not required for Env incorporation in some laboratory cell lines, such as HeLa [9]. Mutational studies support a direct role for the gp41 CT in Env incorporation and recent work has identified potential cellular trafficking proteins which influence Env incorporation in a CT-dependent manner [10], [11]. Early in infection, the gp41 CT has also been reported to stimulate NF-kB, thereby enhancing virus replication in suboptimally activated target cells [12].

Reflecting its diverse roles in the virus replication cycle, mutations in MA can elicit a variety of defects. Single-amino acid mutations have been reported that block Env incorporation [13]–[15]. These mutations are noteworthy in that they typically do not impact any other aspect of the replication cycle and the infectivity block that they impose can be rescued, with the exception of 98EV, by Env glycoproteins bearing short CTs [13], [15], [16]. In 98EV short-tailed Env glycoproteins are incorporated but do not permit infectivity [15]. Mutation of the N-terminal Gly of MA, to which the myristate is covalently attached, or disruption of downstream residues that are either required for Gag myristylation or for the myristyl switch, impair Gag association with membrane [17]–[22]. Mutations in the highly basic patch of residues in the vicinity of residues 17–31 induce Gag retargeting to late endosomes as do mutations in the vicinity of residue 85 [19], [23]. This mistargeting of Gag is thought to result primarily from a loss of MA-PI(4,5)P_2_ binding [2], [5] (for review see [24]). Mutations near residue 50 block virus assembly [19]. All or most of the MA domain can be deleted without blocking virus production if a membrane-targeting signal is attached to the N-terminus of Gag, but such mutants display promiscuous membrane targeting and impaired Env incorporation [25]. Finally, a small number of mutations have been shown to impair an early post-entry step in the replication cycle [26], [27]. These mutations increase Gag-membrane association, suggesting that Gag affinity for membrane is fine-tuned to allow efficient virus assembly and, in the next round, virus entry and uncoating [26], [27].

The mechanism of HIV-1 Env incorporation remains largely obscure. A variety of models have been proposed, including passive/random incorporation, co-trafficking of Gag and Env to a common site of assembly, direct interaction between Gag and Env, and an indirect interaction bridged by a cellular co-factor (reviewed [6], [7]). A central role for MA in Env incorporation is supported by a variety of findings, including the rescue of a gp41 CT mutant deficient in Env incorporation by a selected change in MA [10] and numerous examples of point mutations in MA that block Env incorporation but do not otherwise impair particle production [13]–[15]. As mentioned above, these MA mutants can typically be pseudotyped with alternative envelope glycoproteins that bear short CTs; e.g., murine leukemia virus (MLV) Env, vesicular stomatitis virus (VSV) G glycoprotein, or HIV-1 Env mutants encoding a truncated gp41 CT [13], [16]. These results confirm the original defect as one of Env incorporation and provide further evidence that there may be an interaction between the gp41 CT and MA – the domain of Gag proximal to the membrane and therefore best placed to interact with Env. However, neither the structure of the gp41 CT nor the structure of MA in the context of the virion has been established. The topology of the ∼150 amino acid gp41 CT with respect to the membrane has been the subject of controversy [28]–[30] (for reviews see [8], [31]); it is thus currently unclear how much of the CT is exposed to MA on the cytosolic face of the membrane or what structure(s) the CT adopts.

**Figure 1:**
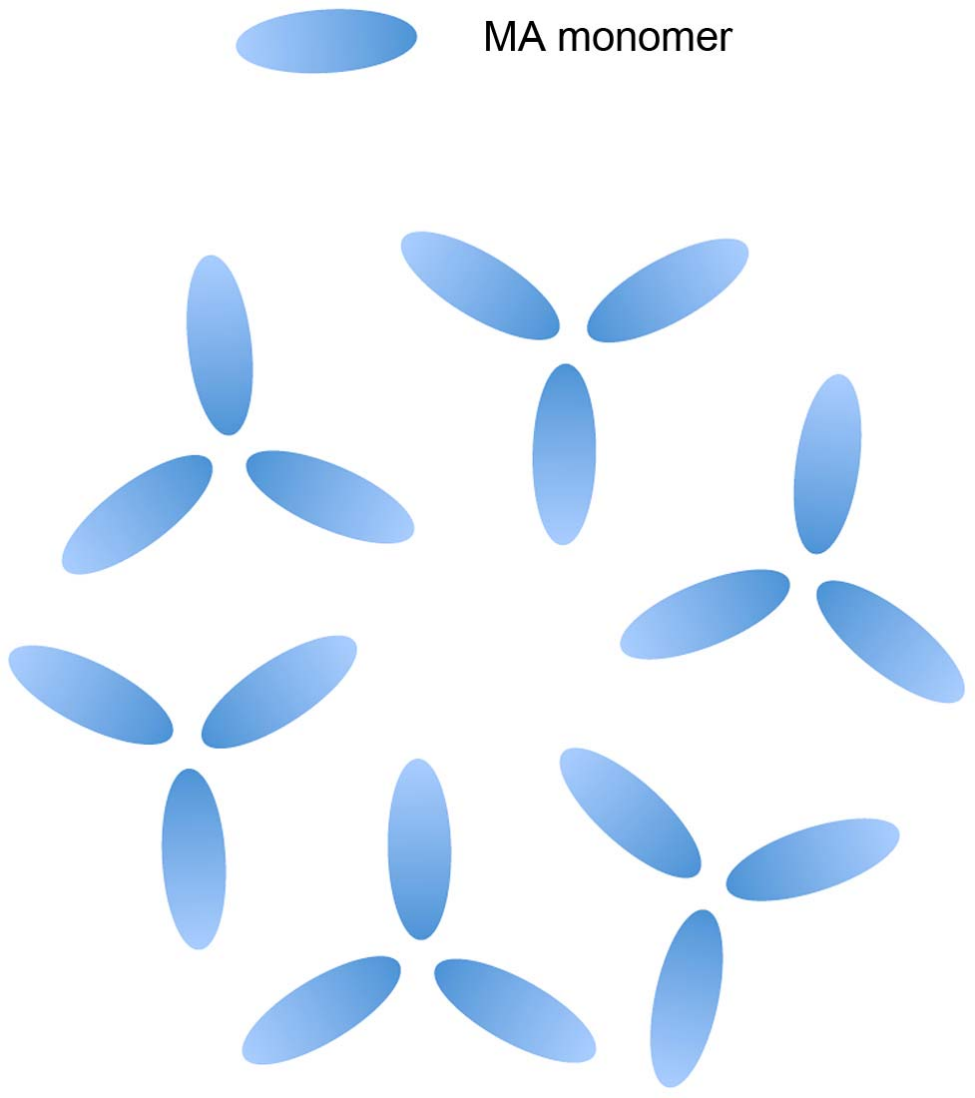
Schematic of MA on an artificial PI(4,5)P_2_ containing membrane. MA arrangement as a hexamer of trimers based on the data published in Alfadhli *et al.* (2009) [35].

Relative to the gp41 CT, more is known about the structure of MA, as high-resolution structures have been generated by solution NMR and X-ray crystallography [32]–[34]. MA adopts a similar conformation in the NMR and crystal structures, but in the NMR structure MA is monomeric whereas MA forms a trimer in crystals [32], [34]–[36]. Recent work has shown that MA organizes into hexamers of trimers on PI(4,5)P_2_-containing membranes *in vitro* [35] (illustrated schematically in Fig. 1). However, evidence for functional MA trimers in infected cells or virions is lacking, and cryo-electron tomography of mature and immature virions has likewise been unable to discern any long-range order within the layer of electron density corresponding to MA. There have been reports of direct interaction between Env and Gag in HIV-1 and simian immunodeficiency virus (SIV) systems; however, these results have proven difficult to reproduce and the nature of the putative Gag-Env interaction remains controversial [37]–[39].

In this study, we identify and characterize the mechanism of action of a MA substitution that is able to rescue a broad range of Env incorporation-defective mutants. Our data suggest that rescue depends on interactions between MA monomers at the trimer interface, providing evidence for the functional relevance of MA trimers in the immature Gag lattice. We propose that the trimeric arrangement of the MA domain of Pr55^Gag^ plays an important role in HIV-1 Env glycoprotein incorporation into virus particles.

## Results

### The 62QR MA mutation rescues the Env incorporation defect exhibited by numerous MA and Env mutants

**Figure 2:**
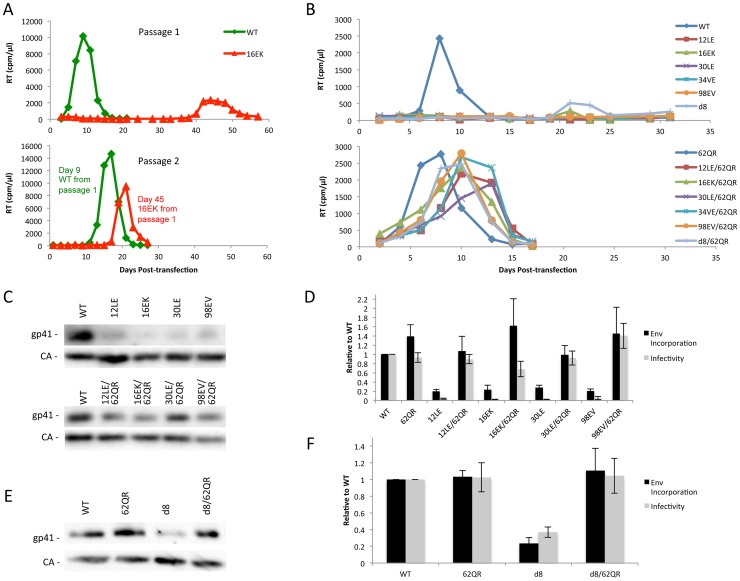
Identification of a second-site mutant capable of rescuing diverse Env-incorporation defective mutants. (A) Jurkat cells were transfected with the indicated molecular clones. At 2-day intervals the cells were split and samples of media were assayed for RT activity. Virus from the WT and 16EK peaks was normalized by RT then used to infect naïve Jurkat cells and replication of the second passage was followed as described above. Genomic DNA was extracted from cells at the time of peak replication in the 16EK samples after both first and second passage cultures, and the MA coding region was amplified by PCR and subjected to DNA sequencing, revealing the original (16EK) and second-site compensatory (62QR) mutations. (B) Jurkat cells were transfected with the indicated molecular clones and replication was monitored as in (A). (C+E) 293T cells were transfected with the indicated molecular clones. At 24 h, supernatants were filtered then virions were pelleted, lysed, and probed by western blotting for gp41 and CA. (D+F) Supernatants were harvested and assayed for infectivity as described in Materials and Methods. Env incorporation was determined as described in Materials and Methods. Infectivity and Env incorporation are expressed relative to the WT value. n = 3, +/− SEM.

Previous studies demonstrated that a MA mutant, 34VE, is unable to incorporate Env into particles and consequently is unable to replicate efficiently in culture [14], [20]. Prolonged culture of the 34VE mutant resulted in the acquisition of a second-site compensatory mutation in MA, 62QR, which reversed the Env incorporation, infectivity and replication defects of 34VE. In more recent analysis, we observed that passaging in the Jurkat T-cell line of another Env-incorporation-deficient MA mutant, 16EK [40], again resulted in the acquisition of 62QR as a compensatory mutation (Fig. 2A). To determine how broadly the 62QR mutation could rescue Env incorporation defects, we combined 62QR with a panel of mutations in MA and Env previously shown to block Env incorporation into particles. These included the MA mutations 12LE, 30LE, 98EV, and the gp41 d8 mutation, a five-amino-acid deletion in one of the helical domains of the gp41 CT [10], [13], [15]. The mutant molecular clones were transfected into Jurkat cells and virus replication was monitored. Each of the single MA mutants that had been previously identified as deficient for Env incorporation either failed to replicate or replicated with a significant delay relative to WT. By contrast, the MA double-mutant clones carrying 62QR all replicated with kinetics similar to those of the WT (Fig. 2B). Perhaps most strikingly, 62QR was also able to rescue the replication defect induced by the d8 deletion in the gp41 CT (Fig. 2B). To confirm that the loss of replication observed with the single mutants was due to loss of infectivity we performed single-cycle infectivity assays using the TZM-bl indicator cell line [41]. These experiments confirmed that the single-mutant viruses were unable to infect TZM-bl cells, whereas the double-mutant viruses carrying 62QR infected TZM-bl cells with an efficiency comparable to that of the WT (Fig. 2D and F). Finally, to confirm that the lack of infectivity was due to loss of Env incorporation and that rescue by 62QR involved the restoration of Env incorporation, we collected viruses from transfected 293T cells and analyzed them by western blotting for the capsid (CA) protein and gp41. Each of the single mutants displayed a lack of gp41 relative to WT, and in each case, double-mutant virions carrying 62QR contained WT levels of gp41 (Fig. 2C and E). Collectively, these data demonstrate that all of the defective mutants tested can be rescued by 62QR, suggesting that their Env incorporation defects are caused by a similar mechanism.

### 62QR Gag is resistant to dominant-negative inhibition by Env-incorporation-deficient Gag

**Figure 3:**
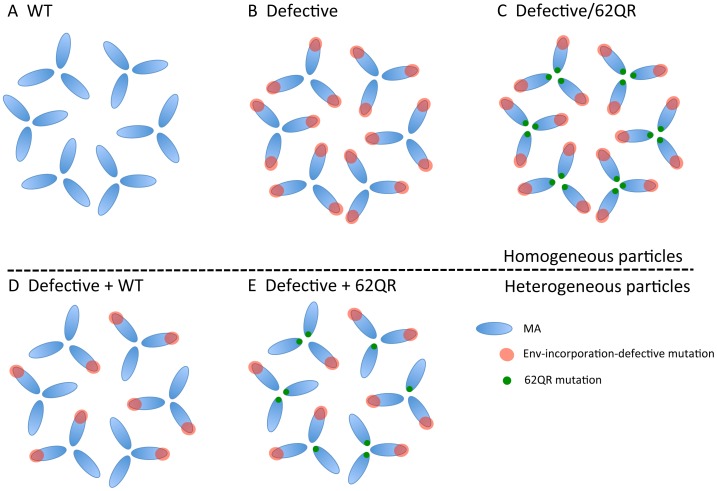
Schematic of MA monomers (blue), organized into a hexamer of trimers, adapted from Alfadhli *et al.* Virology (2009) [**35**]. Under normal circumstances the Gag molecules in a particle are homogeneous, all possessing the same sequence (WT or mutant). To examine phenotypic dominance between the WT Gag, Env-incorporation-defective mutants, and 62QR, heterogeneous particles were produced by co-transfecting two proviral DNAs. The hypothetical MA arrangements are indicated as follows: (A) WT MA. (B) The Env-incorporation-defective mutations (red) cluster at the tips of the MA trimer. (C) The location of the Env-incorporation-defective mutations is indicated as for (B); the green circle near the trimer interface indicates the compensatory mutation 62QR. (D+E) Heterogeneous particles based on a 1∶1 mix of either WT with a defective mutant (D) or 62QR with a defective mutant (E). By contrast with the homogeneous particles (A–C), in D and E each MA molecule possesses a maximum of one mutation, it may be either defective or 62QR, but not both.

**Figure 4:**
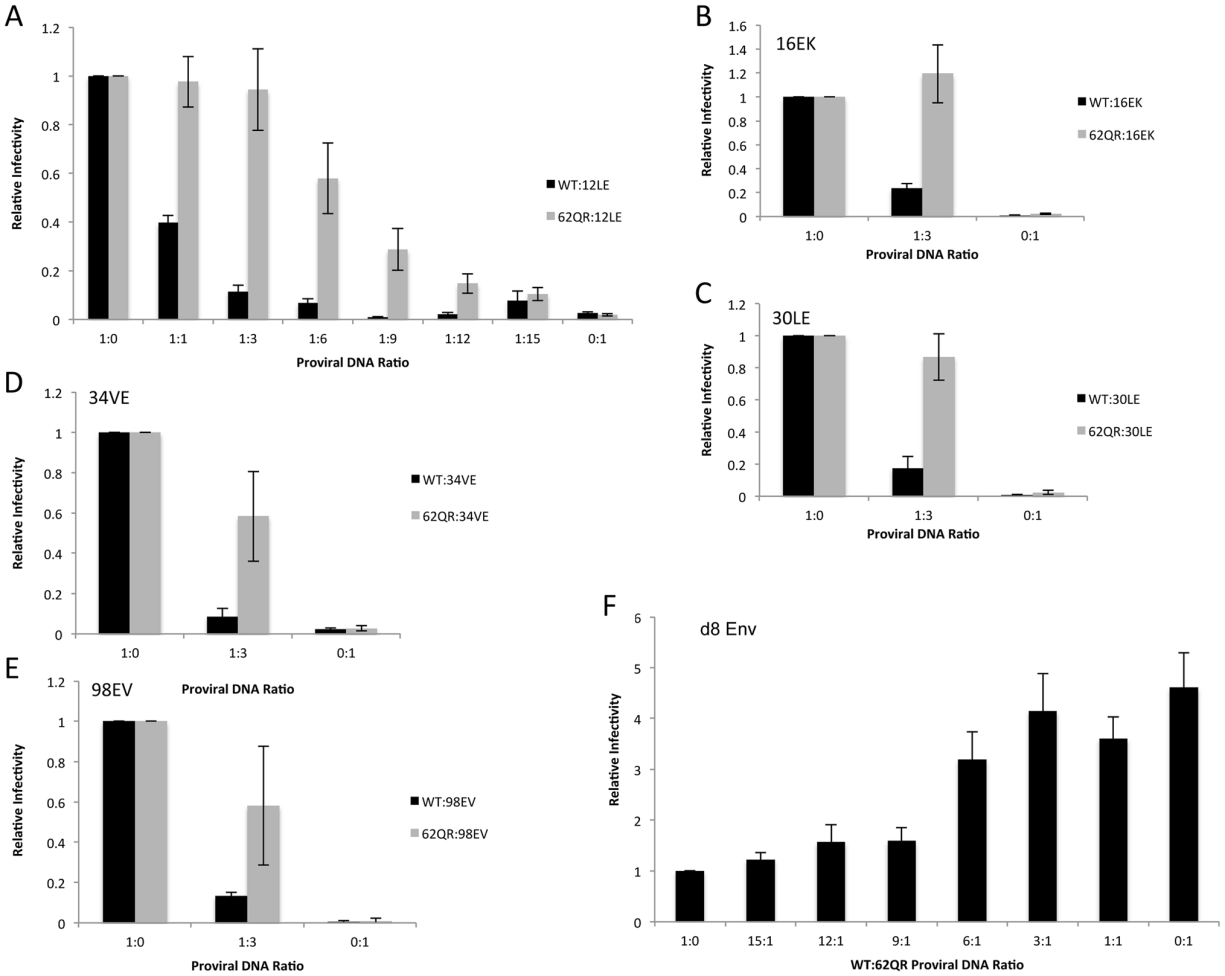
62QR is resistant to dominant-negative inhibition by defective Gag mutants in heterogeneous particles. (A) 293T cells were co-transfected with molecular clones expressing WT or 62QR Gag with the 12LE molecular clone in the ratios indicated (µg∶µg of DNA). At 24 h, supernatants were harvested and assayed for infectivity as described in the Materials and Methods. Infectivity is expressed relative to the WT value. n = 4, +/− SEM. (B–E) 293T cells were co-transfected with molecular clones expressing WT or 62QR Gag with Env-incorporation-defective Gag in the ratios indicated (µg∶µg of DNA). Infectivity relative to WT was determined as described for (A). n = 3, +/− SEM. (F) 293T cells were co-transfected with molecular clones expressing WT or 62QR Gag with d8 gp41 in the ratios indicated (µg∶µg of DNA). Infectivity relative to WT was determined as described for (A) n = 4, +/− SEM.

To address the mechanism by which 62QR rescues Env incorporation we examined Env incorporation into heterogeneous virus particles. A prevailing hypothesis for Env incorporation into HIV-1 particles posits a direct Gag-Env interaction [6]. If this were the mechanism of recruitment, then by making particles with a range of ratios of Env-recruiting (e.g., WT or 62QR) and Env-excluding (e.g., 12LE) Gags we should see Env incorporation vary in proportion to the amount of Env-recruiting Gag in the particle; no difference between WT and 62QR would be expected in this context (a schematic representation of homogeneous and heterogeneous particles is shown in Fig. 3). This experiment was performed in parallel with WT plus 12LE and 62QR plus 12LE molecular clones. As an increasing amount of the 12LE molecular clone was cotransfected with the WT clone, a rapid decline in virus infectivity was observed. By contrast, when 12LE and 62QR clones were cotransfected at the same ratios the effect was much less pronounced (Fig. 4A). Indeed, even at a 3-fold excess of 12LE over 62QR, infectivity of 62QR:12LE virus was unaffected. It was not until 12LE was present at a 9-fold excess over 62QR that 62QR:12LE virus infectivity was comparable to that of virus produced at a 1∶1 WT∶12LE ratio. Similar analyses were performed, using a more limited range of input DNA ratios, with the mutants 16EK, 30LE, 34VE and 98EV with comparable results (Fig. 4B–E). We performed an analogous set of experiments using the d8 gp41 mutant [10]. WT and 62QR molecular clones, both expressing the d8 Env mutant, were cotransfected over a range of DNA ratios. As shown in Fig. 4F, particles produced with WT Gag are poorly infectious; virions produced by 62QR Gag in the context of d8 Env are highly infectious. Even when WT DNA input was in 3-fold excess over 62QR, virus infectivity was not reduced by the d8 Env mutation (Fig. 4F). It was not until WT was present in six-fold excess over 62QR that infectivity was reduced below 50% of that measured with 62QR alone (Fig. 4F). These data demonstrate that the 62QR mutant, even when present at relatively low levels, is able to rescue, *in trans*, infectivity defects imposed by mutations in MA or the gp41 CT.

### Most mutations at MA residue 62 are tolerated but do not affect Env incorporation

**Figure 5:**
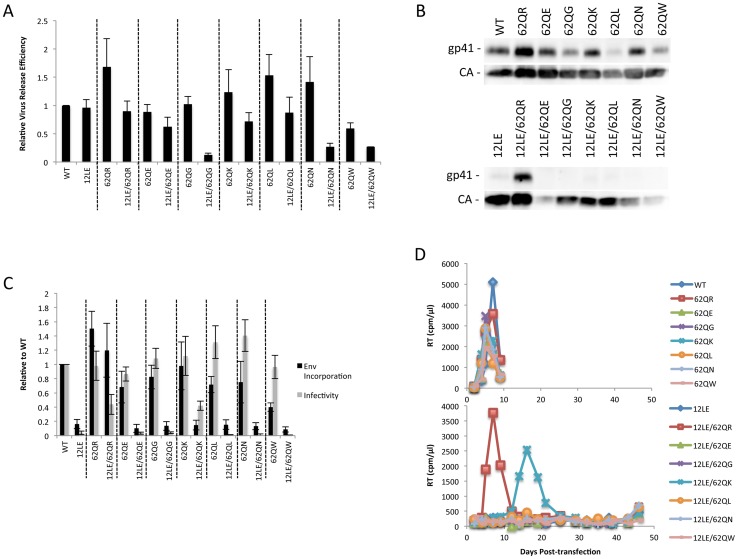
Vertical scanning of MA residue 62 to determine effects on Env incorporation and ability to rescue Env-incorporation-defective mutants. (A) HeLa cells were transfected with the molecular clones indicated. Virus release efficiency was determined by metabolic labeling with ^35^S[Met/Cys] as described in Materials and Methods. n = 3, +/− SEM. (B) 293T cells were transfected with the indicated molecular clones. At 24 h, supernatants were filtered then virions pelleted, lysed, and probed by western blotting for gp41 and CA. (C) Supernatants from (B) were harvested and assayed for infectivity as described in Materials and Methods. Env incorporation was determined as described in Materials and Methods. Infectivity and Env incorporation are expressed relative to the WT value. n = 6, +/− SEM. (D) Jurkat cells were transfected with the indicated molecular clones. At 2-day intervals the cells were split and samples of media were assayed for RT activity.

Residue 62 lies in a region of MA that has not been previously implicated in Env incorporation; we therefore performed vertical scanning mutagenesis at this position to gain insight into its role in Env incorporation and the rescue of incorporation-defective mutants. We generated six additional mutants, 62Q[E/G/K/L/N/W], and in parallel introduced each of these substitutions in the context of 12LE to look for rescue of the 12LE-imposed defect in Env incorporation. None of the single mutations at position 62 severely impaired virus release, infectivity or Env incorporation (Fig. 5A, B and C). The most severe defect was seen in 62QW, which displayed approximately 50% reductions in Gag release and Env incorporation. All mutants replicated with WT kinetics in Jurkat cells (Fig. 5D).

The 12LE/62Q[E/G/K/L/N/W] double mutants were subjected to the same analyses described above for 12LE/62QR. Impaired virus release efficiency was observed for the double mutants 12LE/62QG, 12LE/62QN and 12LE/62QW. This defect in particle production is most likely due to an adverse effect on MA folding of introducing both 12LE and 62QG/N/W mutations, though we cannot exclude the possibility that these double mutants could be mistargeted. Unlike 62QR, none of the other residue 62 mutants was able to fully rescue the virus replication, infectivity, and Env incorporation defects imposed by 12LE (Fig. 5). The 12LE/62QK mutant exhibited a partial rescue, as infectivity in TZM-bl cells was comparable to that of 12LE/62QR (Fig. 5C), and 12LE/62QK replicated sooner than the 12LE/62Q(E/G/L/N/W] double mutants in Jurkat cells (Fig. 5D). However, replication was delayed relative to that of the WT (Fig. 5D) and no rescue of Env incorporation was apparent (Fig. 5B and C). Virus recovered from the 12LE/62QK cultures in these experiments had acquired 34VI or 34VL. 34VI is a previously characterized MA mutation that is capable of rescuing the Env incorporation and replication defects imposed by 12LE and d8 [10], [14]. These data demonstrate that residue 62Q is not crucial for Env incorporation in the context of otherwise-WT MA, and that 62QR is unique among the mutants analyzed for its ability to fully rescue Env incorporation defects.

### Inter-subunit interactions in the MA trimer are required to rescue Env incorporation

**Figure 6:**
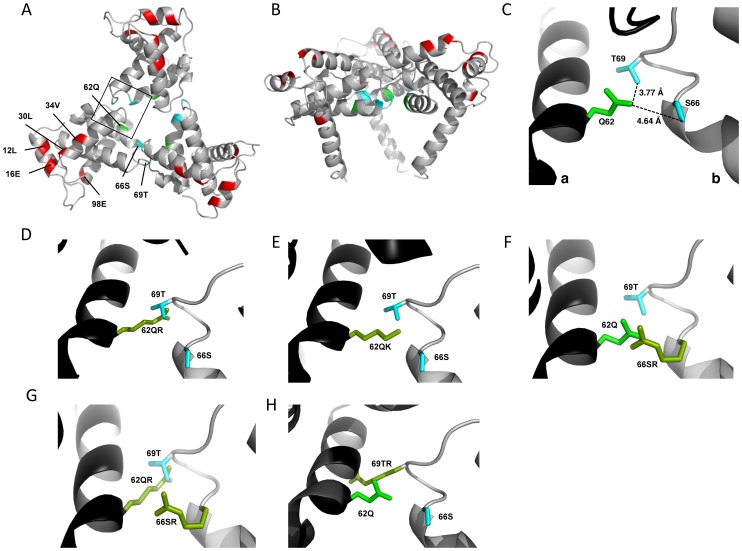
Potential for intersubunit interactions in the MA trimer. MA trimer as described in Hill *et al.* PNAS (1996), showing (A) a top-down view and (B) a side-on view [32]. Env incorporation defects, red; Q62, green; Ser66 and Thr69, cyan. (C) Close-up view of boxed area from (A), showing Q62 side chain (green), and the side chains of S66 and T69 (cyan) of a second MA monomer. Chain a, black; chain b, gray. Distances between the oxygen atoms of Q62 carbonyl group and the S66 and T69 hydroxyl groups are indicated. Modeled configurations for R62 (D) K62 (E), R66 (F), R66, in combination with R62 (G) and R69 (H). Mutagenesis and rendering performed using MacPymol [70].

**Figure 7:**
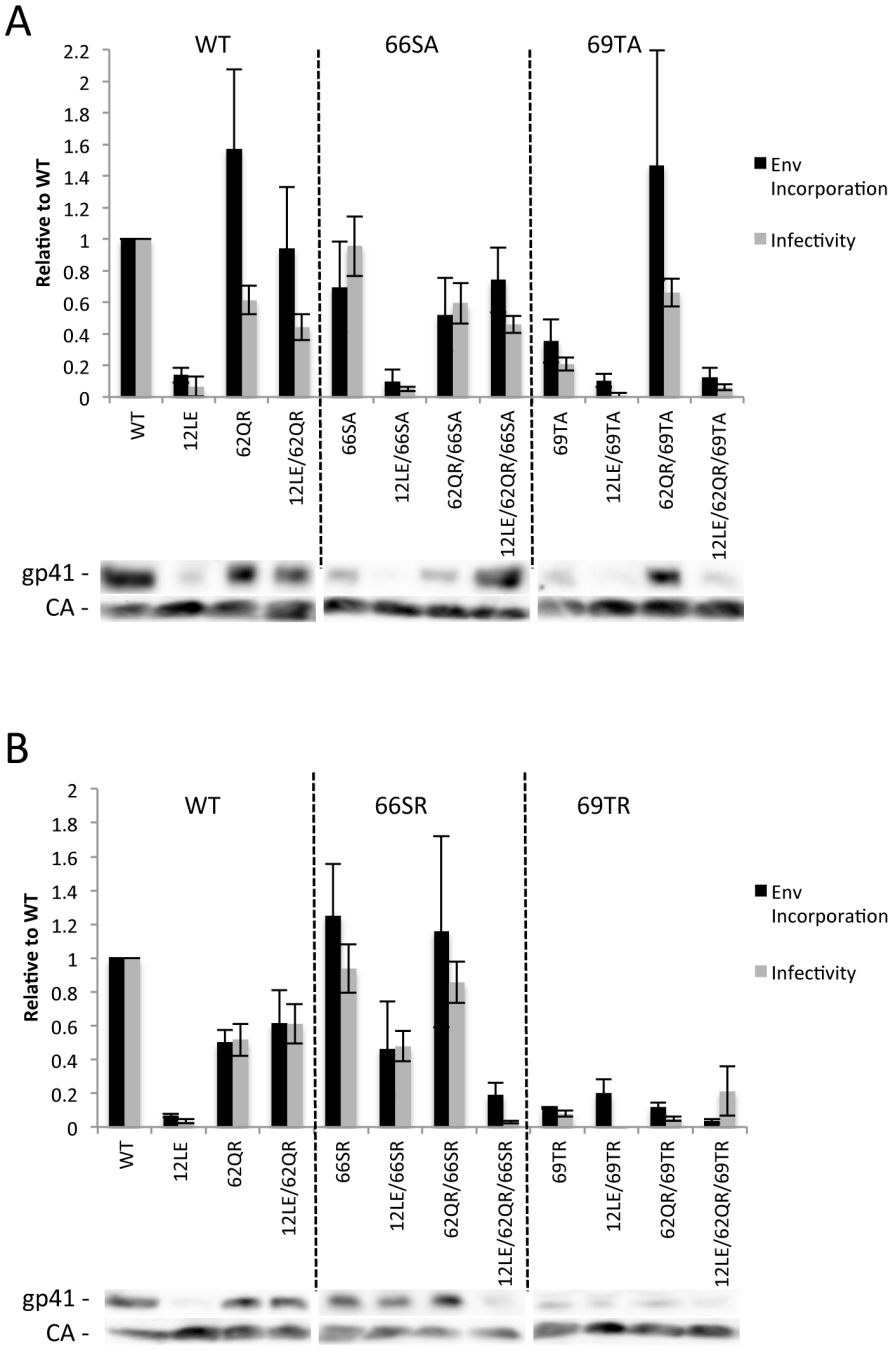
The effect of mutations at the trimer interface on rescue of Env incorporation. 293T cells were transfected with the indicated molecular clones. At 24Materials and Methods. Infectivity is expressed relative to the WT value. Supernatants were also filtered and virions pelleted, lysed, and probed by western blotting for gp41 and CA. Env incorporation was determined as described in Materials and Methods and is indicated relative to WT. Representative blots are shown below each graph. n = 5–7, +/− SEM. (A) Ala mutants of S66 and T69. (B) Arg mutants of S66 and T69.

**Figure 8:**
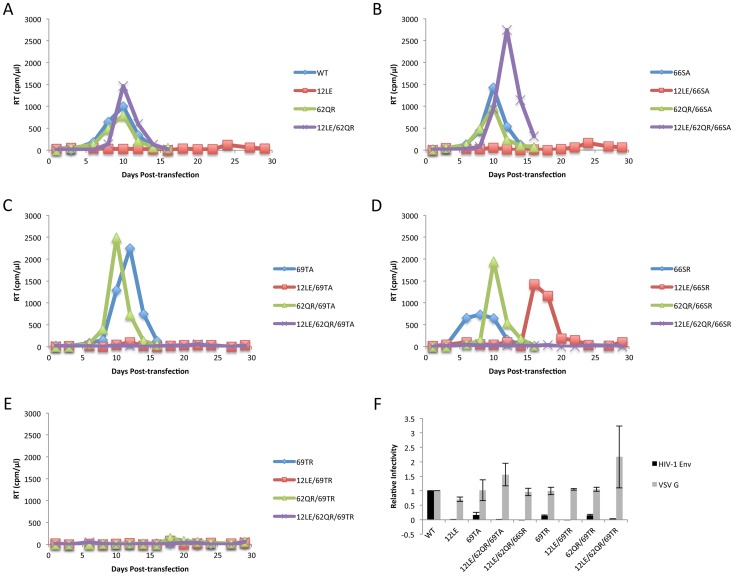
Replication of S66 and T69 mutants in Jurkat cells. Jurkat cells were transfected with the indicated molecular clones. At 2-day intervals the cells were split and samples of media were assayed for RT activity. In each graph of WT pNL4-3, 12LE, 62QR or 12LE/62QR mutations are combined with (A) WT; (B) 66SA; (C) 69TA; (D) 66SR; (E) 69TR. (F) 293T cells were co-transfected with the indicated molecular clones and vectors expressing HIV-1 Env or VSV-G. At 24 h, supernatants were harvested and assayed for infectivity as described in Materials and Methods. n = 3, +/− SEM.

The positions of the MA mutations that block Env incorporation were identified on the previously published MA crystal structures (Fig. 6A and B; Fig. 3) [32], [35]. The mutations in MA that affect incorporation of Env cluster towards the tips of the arms of the MA trimer; by contrast, the rescue mutation at position 62 is located near the center. As the gap in the MA lattice at the three-fold axis is relatively small and residue 62 is not prominently exposed to the membrane it seemed improbable that this site was engaging in direct contact with the gp41 CT (Fig. 6A and B – in 6B the membrane would be at the top of the structure [5]). Instead, we hypothesized that the rescue depended on altering the structure of the MA lattice via novel interactions. The closest side chains to Q62 of chain a (Fig. 6C) are those of S66 and T69 in chain b; although these residues have polar side chains, the crystal structure indicates that they are too distant from Q62 to form hydrogen bonds, which typically require less than 3 Å between the nucleophilic atoms [42] (Fig. 6C). It is possible, however, that when Q62 is replaced by R62, a longer side chain combined with the greater positive charge may permit inter-subunit interactions to occur, perhaps with T69 (Fig. 6D). A similar possibility exists with 62QK, although our data suggest it may be a less favored configuration (Fig. 4; Fig. 6D and E). To test this hypothesis, the residues at positions 66 and 69 were mutated to Ala in the context of WT, 12LE, 62QR and 12LE/62QR. The 66SA mutation had no effect on the phenotypes of the four viral clones (Fig. 7A). By contrast, 69TA blocked the ability of 62QR to rescue 12LE infectivity and Env incorporation, although 62QR/69TA was as infectious as 62QR alone (Fig. 7A). 69TA as a single mutant was also impaired for infectivity and Env incorporation, suggesting that residue 69 may be involved in MA function in the WT molecule (Fig. 7A). 69TA also showed a small (2-day) delay in replication (Fig. 8C), consistent with its reduced Env incorporation and single-cycle infectivity (Fig. 7A). The partial defect of 69TA was relieved by 62QR (Fig. 7A; Fig. 8C). To further examine the role of interactions in this region, an additional panel of mutants was generated by changing S66 and T69 to Arg. Strikingly, 66SR behaved like 62QR in its ability to rescue 12LE Env incorporation and infectivity, indicating that an Arg at either residue 62 or 66 could rescue the 12LE defect (Fig. 7B; Fig. 6F). In contrast, combining 12LE, 62QR, and 66SR resulted in a loss of infectivity, suggesting either a loss of interaction or potentially repulsive interactions between R66 and R62 (Fig. 7B; Fig. 6G). All virions bearing 69TR displayed very low infectivity and Env incorporation (Fig. 7B); structural modeling suggests that 69TR may introduce steric hindrance at the MA trimer interface (Fig. 6H). These single-cycle results were confirmed by performing virus replication assays in Jurkat cells (Fig. 8). In each case, mutants that were found to be infectious were also able to replicate, although 12LE/66SR displays a moderate delay relative to WT and, like 12LE/62QK, 12LE/66SR acquires 34VI to permit efficient replication. Those mutants that were neither infectious nor able to replicate in Jurkat cells were pseudotyped with VSV-G (Fig. 8F). The VSV-G pseudotyped particles were infectious in TZM-bl cells, confirming that the infectivity defect related to Env incorporation and not any other aspect of the infectivity process. As expected, these mutants could not be pseudotyped with HIV-1 Env (Fig. 8F), consistent with the data presented above. Collectively, these data illustrate the importance of the MA-MA interface in the rescue of Env-incorporation defective mutants, suggesting that the multimeric arrangement of MA is a critical factor in HIV-1 Env incorporation.

## Discussion

Various models can be invoked to explain the incorporation of Env into HIV-1 particles, the principle unresolved issues being whether or not Env is actively recruited into virions and if so, whether Env interacts directly with Gag or indirectly via a bridging cellular factor [6]. Evidence to support active recruitment is provided by five observations. First, the existence of mutations in MA and Env that prevent Env incorporation is consistent with a direct recruitment and suggests the presence of interacting motifs, although it does not address the question of whether the interaction is direct or indirect. Secondly, it has been reported that HIV-1 Env is retained on immature particles even after removal of the viral membrane with detergent [43]. This retention is dependent on the long gp41 CT, again consistent with an interaction between the CT and Gag. Third, there have been reports of interaction between Gag and Env in cells and with recombinant proteins *in vitro* [37]–[39]; however, this interaction has been difficult to demonstrate consistently. Fourth, Env has been reported to influence the site of virus budding in polarized epithelial cells and T cells [44], [45]. Finally, the observation that Gag processing (virus maturation) affects Env fusogenicity is consistent with cross-talk between Gag and the CT of gp41 [46]–[48].

In light of the difficulties encountered in reproducibly demonstrating a direct interaction between MA and Env, we sought an alternative approach to determine whether rescue by 62QR conformed to a model of Env recruitment via MA binding. We initially observed rescue in homogeneous particles in which all MA molecules contain both the defective and rescue mutations. If the defective mutants fail to incorporate Env because MA cannot bind the gp41 CT, then in particles composed of a mixture of Env-recruiting MA (WT or 62QR) and non-recruiting MA (the defective mutants) Env incorporation should vary in proportion to the amount of Env-recruiting MA the particle contains. In this scenario, WT and 62QR would function similarly in the heterogeneous particle assay; both should gradually become less infectious as a defective mutant is added to the particles. This was not observed. Rather, we observed a “dominant-positive” effect whereby particles containing 62QR retained infectivity and Env incorporation even when 62QR MA was a minority population compared to the defective mutant. WT-containing particles did not exhibit this phenotype, supporting the hypothesis that 62QR MA establishes a Gag structure that is more accommodating of the long gp41 CT.

Although the structure of the gp41 CT is currently unknown, structures have been solved for MA [32], [36]. The NMR structure shows an MA monomer, whereas the crystallographic structure reveals a trimeric arrangement. In the context of either structure the defective mutations are clustered, indicating the surface of MA that is important for Env packaging. If this site on MA actively binds Env then the rescue mutation might be expected to be nearby, and effect rescue by creating a new interaction to replace that lost by the defective mutant. Contrary to this hypothesis, 62QR is located at a site distant from the original mutations. Alternatively, 62QR could create an entirely new Env-binding site on the opposite side of MA. This hypothesis also faces multiple challenges. First, in the trimeric structure of MA, 62QR lies at the interface of the trimer, providing a much smaller gap in the MA lattice than that found in the center of the MA hexamer of trimers [35] (Fig. 3). Second, residue 62 is not present on the face of MA that would be expected to oppose the PM, so would require part of the gp41 CT to extend into the trimeric interface. In such a scenario it is hard to explain the lack of inhibitory phenotype of the non-conservative 62Q mutations reported here. Third, 62QR is able to rescue d8, an Env mutant with a small deletion that is not efficiently packaged into WT particles. If this Env mutant had lost a MA-interacting motif then 62QR would be required to interact with an entirely new surface on Env. Our results do not exclude the possibility of a MA-Env interaction, but they suggest that it may not be required for Env recruitment into virions.

The crystal structures of HIV-1 and SIV MA provide evidence for the existence of a MA trimer, and trimers of MA and Gag have been observed with recombinant proteins *in vitro*, although this trimer has not been observed directly in cells or virions [32], [34], [49], [50]. The trimer hypothesis gained recent support from a lower-resolution approach that examined a more physiologically relevant two-dimensional array of MA on a phospholipid membrane. This analysis initially revealed MA hexamers, but when membranes containing PI(4,5)P_2_ were used, MA arranged itself as hexamers of trimers [35], [51] (Fig. 8). In addition to supporting the trimer structure observed in the earlier crystals, these findings reveal a higher-order structure with potential relevance to Env packaging. The mutations that block Env incorporation cluster on the tips of the spokes of the MA trimer; in the context of the hexamer of trimers, this places them around the edge of a large hole in the proposed MA lattice (Fig. 3). It could be envisaged that the gp41 CT fits into this hole during particle assembly, and that the defective mutants are unable to package Env due to steric hindrance and/or charge repulsion resulting from mutations around the perimeter of this hole or mutations in gp41 CT, such as d8, that may alter both its shape and orientation. This type of defect would explain the ability to packaged short tailed envelopes and could be rescued by a distant mutation if it were able to modify the structure of the MA lattice; 62QR is located near the trimer interface of the MA crystal structure and is therefore ideally placed to impose such a long-range change. The model that 62QR modifies the structure of the MA lattice could also explain the different phenotype of WT and 62QR in mixed Gag particles.

Detailed examination of the crystal structure of the MA lattice revealed two potential partners for 62QR in forming inter-subunit interactions: S66 and T69. These polar residues are too distant from Q62 to form hydrogen bonds, albeit only by 1–2 Å. The replacement of Gln with Arg in 62QR introduces a larger, charged side chain; if this interaction were sufficient to alter the structure of the MA lattice it could relieve the steric hindrance introduced by the defective mutations. Our data support this hypothesis, as mutation 69TA blocks the ability of 62QR to rescue 12LE, while other combinations of residues that could form interactions between the subunits, including 62Q with 66SR, are able to rescue Env incorporation and infectivity. Furthermore, introduction of multiple positively charged residues blocks rescue of 12LE, even where both mutations (62QR and 66SR) are independently capable of rescue. This underscores the likelihood that the critical interaction is taking place between MA monomers and loss of rescue is due to the loss of intersubunit interactions.

The apparent importance of interactions between MA monomers in the MA trimer over primary amino acid sequence raises important questions about the role of MA trimerization in HIV-1 Env incorporation. The putative first step in Env incorporation is the trafficking of both Gag and Env to lipid microdomains where assembly occurs [2], [52]–[55]. These domains may be characterized by physical and biochemical features such as membrane curvature, distinct lipid and protein composition, and the presence of Gag itself [3], [56]–[60]. These factors likely permit Env clustering at assembly sites without direct interaction with Gag, as foreign Env molecules have also been reported to cluster at budding sites [61]. Recent studies using super-resolution microscopy techniques demonstrate that Gag induces HIV-1 Env clustering and reduced Env mobility at sites of Gag assembly [62], [63]. These effects of Gag assembly on Env clustering and mobility are largely dependent on the gp41 CT [62], [63]. Mutations in MA that disrupt Env incorporation reverse the Gag-induced Env clustering [62]. Interestingly, Env clustering could be visualized in regions peripheral to Gag assembly sites, suggesting that clustering was not solely due to Gag-Env interaction but was also likely influenced by the formation of a Gag-induced microdomain that favored Env retention near the budding site [62]. Defects in Env incorporation induced by mutations in MA likely arise as a result of steric exclusion of Env from the assembled Gag lattice rather than a lack of recruitment to the budding site. Likewise, mutations in the gp41 CT (e.g., d8) probably alter the structure of the gp41 CT such that it is excluded from the Gag lattice. This steric exclusion model is supported by the observation that mutations in MA and gp41 such as those analyzed here block Env incorporation even in cell types such as HeLa that do not require the long gp41 CT for incorporation [9], [10]. Also consistent with this hypothesis, large or charged side chains at position 12 in MA impair Env incorporation more dramatically than smaller, hydrophobic side chains [14]. If there is insufficient space in the Gag lattice to accommodate the gp41 CT, Env may be excluded and will diffuse away from the budding site.

**Figure 9:**
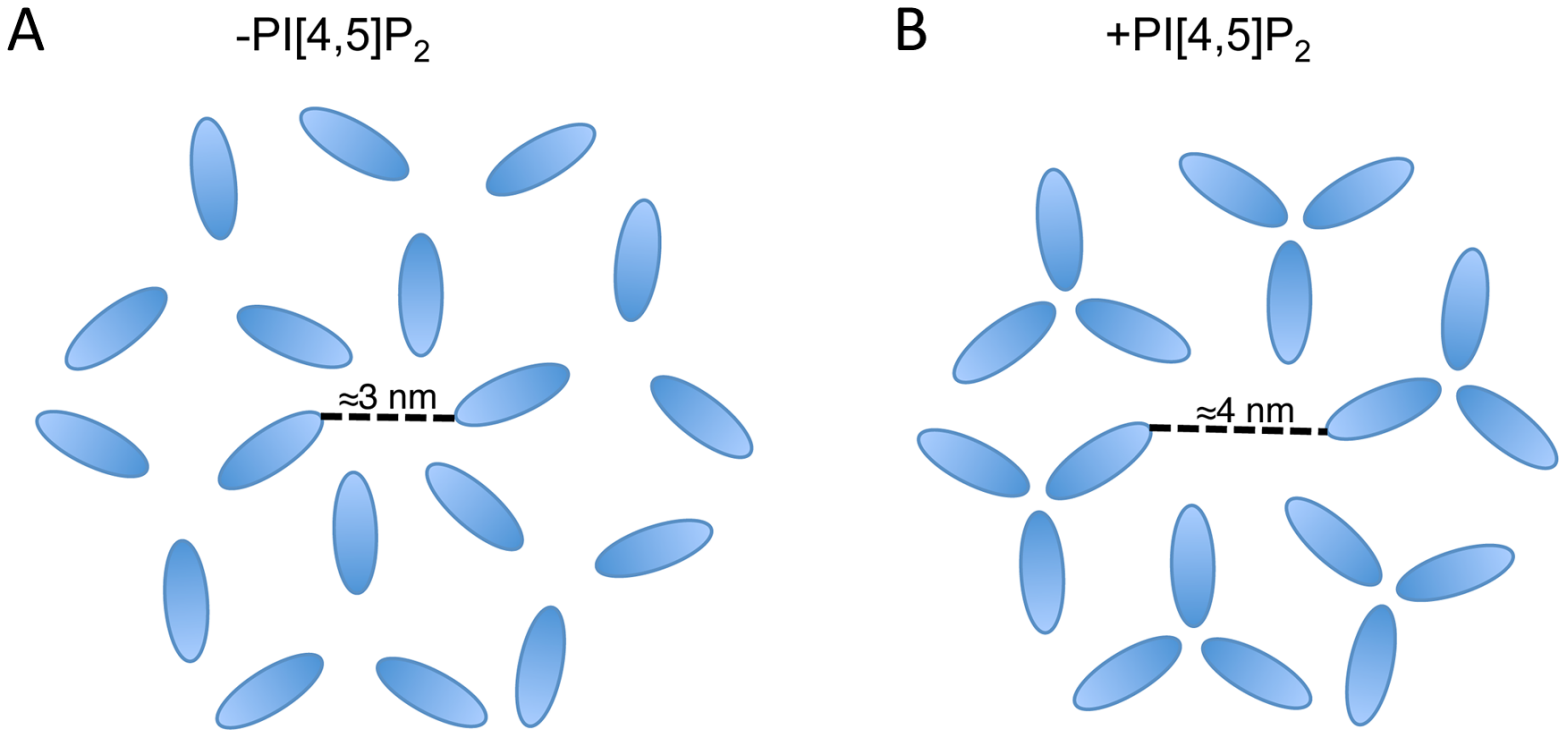
Schematic of two possible MA lattices. MA arrangements are based on the data published in Alfadhli *et al.* (2007) and Alfadhli *et al.* (2009) [35], [51]; in those papers CA is also shown forming a hexameric lattice beneath MA. (A) In the absence of PI[4,5]P_2_ MA forms a hexamer reflecting the underlying CA lattice. (B) In the presence of PI[4,5]P_2_ MA forms trimers resulting in a larger space at the center of the hexamer. Based on the location of Env-incorporation defective mutants in MA, the Env CT may be located at the hexameric center. The structure of the Env CT is unknown.

It is notable that the available structures of a 2D MA lattice are broadly similar, either hexameric in the absence of PI(4,5)P_2_ or a hexamer of trimers in the presence of PI(4,5)P_2_ [35], [51]. The structures differ in the extent to which MA monomers pack together as trimers, effectively increasing the diameter of the gap in the center of the hexamer. It is possible that the role of MA trimerization is to create this larger gap in the MA lattice and thereby permit the long gp41 CT to pack into the lattice (Fig. 9). Such a hypothesis may explain the reduced Env incorporation exhibited by 69TR; modeling indicates that such a mutation would cause steric hindrance to the trimer structure, as it is currently understood. Mutations predicted to impair MA trimerization were previously found to reduce infectivity, although that study did not report loss of Env incorporation [64]. Further investigation of 69TR and other mutations that would likely destabilize the MA trimer will be invaluable in understanding the role this structure plays in particle assembly and Env incorporation.

In summary, we have identified a MA mutation capable of global rescue of Env incorporation defects and have investigated the mechanism of rescue. The data reveal the importance of intersubunit interactions in a MA trimer, strongly supporting the existence of this structure in immature virions. Our data also suggest that the inability of several mutants to incorporate Env into particles may be caused by steric hindrance if the gap in the MA lattice is no longer large enough to accommodate the gp41 CT. If this is the case, then two possible drug targets can be proposed. Firstly, the sensitivity of Env incorporation to mutations at the tips of the MA trimer indicates that compounds binding at this site could inhibit Env incorporation. Secondly, the importance of intersubunit interactions within the MA trimer suggests that compounds able to disrupt intertrimer interactions may also inhibit Env incorporation, or potentially other functions of MA. Our data do not exclude the possibility of a direct interaction between MA and Env; they do, however, point to the importance of multimeric MA structure in Env incorporation. Determining the role of the MA trimer in HIV-1 particle assembly and replication remains a critical goal in extending both our understanding of basic retroviral biology and our range of virus-specific therapeutic targets.

## Materials and Methods

### Cells lines and antibodies

HeLa and TZM-bl cells were cultured in Dulbecco's Modified Eagle's Medium (DMEM), supplemented with 5% v/v fetal bovine serum (FBS), 100 U/ml penicillin, 100 µg/ml streptomycin, and 2 mM L-glutamine (Gibco). TZM-bl is a HeLa-derived indicator cell line that expresses luciferase following infection by HIV [65]. 293T cells were cultured in DMEM, supplemented with 10% v/v FBS, 100 U/ml penicillin, 100 µg/ml streptomycin, and 2 mM L-glutamine. Jurkat CD4^+^ T-cells were cultured in Roswell Park Memorial Institute (RPMI) 1640 medium, supplemented with 10% v/v FBS, 100 U/ml penicillin, 100 µg/ml streptomycin, and 2 mM L-glutamine. Anti-HIV-1 IgG is pooled patient serum obtained from the NIH AIDS Reagent Program. HIV-1 gp41 was detected with the 2F5 monoclonal antibody [66].

### Plasmids

HIV-1 particles were generated using the full-length proviral clone pNL4-3 [67]. Point mutations were introduced by first subcloning the *Bss*HI-*Spe*I fragment from pNL4-3 into pBluescript (Stratagene). Mutations were introduced using the Quikchange method (Stratagene) following the manufacturer's instructions and the mutant *Bss*HI-*Spe*I fragment was recloned into pNL4-3. All mutations were confirmed by DNA sequencing (Macrogen). For pseudotyping experiments VSV-G was expressed from pHCMVG, a gift from J. Burns (University of California, San Diego).

### Virus replication, release, and infectivity

HIV-1 replication was assayed by rate of spreading infection in Jurkat cells as reported previously [19]. Virus replication was monitored by measuring RT activity as described [68]. When necessary, genomic DNA was extracted using QIAamp (Qiagen) following the manufacturer's protocol; proviral sequences were amplified by PCR and sequenced (Macrogen). The efficiency of virus particle assembly and release was assayed by radiolabeling newly synthesized Gag then separately immunoprecipitating cell-associated and virion-associated Gag. Immunoprecipitated proteins were separated by SDS-PAGE and quantified using a Personal Molecular Imager (Biorad) [69]. The percentage of the total expressed Gag that is released from the cell as virion-associated material indicates the efficiency of particle release. For infectivity assays, virus-containing supernatants were generated by transfecting subconfluent 293T cells in 12-well plates. Shortly before transfection the medium was removed and replaced with 500 µl DMEM. 1 µg DNA was diluted in 500 µl serum-free DMEM, mixed thoroughly with 7.5 µl polyethyleneimine [1 mg/ml polyethyleneimine (PEI), 20 mM HEPES pH 7.2] and incubated for 30 min at room temperature before dropwise addition to cells. Supernatants were harvested 24–48 h post-transfection and assayed for RT activity as described [68]. TZM-bl cells were infected with the supernatants and the luciferase signal was measured 24 h post-infection using Britelite Plus (Perkin-Elmer). Infectivity was defined as the level of luciferase expressed by TZM-bl cells divided by the total amount of virus (RT) with which they were infected.

### Env incorporation into virions

Virions were harvested 24–48 h post-transfection by filtering supernatant through a 0.45 µm membrane then pelleting by centrifugation at 76,000×g for 1 h at 4°C. Virions were resuspended in 2× Laemmli buffer (120 mM Tris-Cl [pH 6.8], 4% SDS, 20% glycerol, 10% β-mercaptoethanol, 0.02% bromophenol blue) and analyzed by western blotting. Protein samples were separated by SDS-PAGE and transferred to a polyvinylidene fluoride (PVDF) membrane (Immobilon, Millipore) by semi-dry electroblotting. Membranes were probed with primary antibody overnight at 4°C, washed, then incubated for 1 h with species-specific horseradish peroxidase-conjugated secondary antibody. After the final washes, bands were revealed by chemiluminescence; working-solution was produced by mixing an equal volume of solution 1 [25 mM Luminol (3-aminophthalydazide), 0.3 mM p-coumeric acid, 100 mM Tris-HCl, pH 8.5] with solution 2 (0.01% hydrogen peroxide, 100 mM Tris-HCl, pH 8.5). After incubation for 1 min at room temperature, membranes were exposed to a charge-coupled device in a Universal Hood II (Biorad). For weak signals, Supersignal West Femto reagent (Thermo Scientific) was used following the manufacturer's instructions. Quantification was performed using ImageLab software (Biorad). To calculate Env incorporation, volumes (average pixel intensity multiplied by the area covered by the band) were determined for each gp41 band and divided by the volume for the corresponding CA band.
